# LARP1 isoform expression in human cancer cell lines

**DOI:** 10.1080/15476286.2020.1744320

**Published:** 2020-04-14

**Authors:** Hagen Schwenzer, Mai Abdel Mouti, Pia Neubert, Josephine Morris, Joanne Stockton, Sarah Bonham, Martin Fellermeyer, James Chettle, Roman Fischer, Andrew D. Beggs, Sarah P. Blagden

**Affiliations:** a Department of Oncology, University of Oxford, Oxford, UK; b Institute of Cancer & Genomic Sciences, University of Birmingham, Birmingham, UK; c Target Discovery Institute, Nuffield Department of Medicine, University of Oxford, Oxford, UK

**Keywords:** LARP1, RNA Binding Proteins, cancer, alternative protein isoforms

## Abstract

LARP1 is an oncogenic RNA-binding protein required for ribosome biogenesis and cancer cell survival. From published *in vitro* studies, there is disparity over which of two different LARP1 protein isoforms (termed the long LI-LARP1 and short SI-LARP1) is the canonical. Here, after conducting a series of biochemical and cellular assays, we conclude that LI-LARP1 (NM_033551.3 > NP_056130.2) is the dominantly expressed form. We observe that SI-LARP1 (NM_015315.5> NP_056130.2) is epigenetically repressed and that this repression is evolutionarily conserved in all but a small subclade of mammalian species. As with other LARP family members, there are multiple potential LARP1 mRNA isoforms that appear to be censored within the nucleus. The capacity of the cell to modulate splicing and expression of these apparently ‘redundant’ mRNAs hints at contextually specific mechanisms of LARP1 expression.

## Introduction

The La-related proteins (LARP) are an evolutionarily conserved family of RNA-binding proteins (comprised of LARP 1, 1B, 3, 4A, 4B, 6 and 7) linked to cancer [1]. LARP1 is oncogenic with higher tumour levels of LARP1 protein corresponding with adverse prognosis in ovarian, colorectal and prostate cancer [2–4]. LARP1 is believed to bind mRNA at two sites; via its conserved La module, an RNA-binding region comprised a La domain and an adjacent RNA recognition motif (RRM) and via its C terminal ‘DM15 domain’ [5]. LARP1 is complexed with poly-A binding protein (PABP) and binds an interactome of over 3000 mRNAs including 5ʹTOP-motif containing transcripts which are known to encode the ribosomal machinery [3]. As it has been shown to be phospho-regulated by mTORC1, LARP1 has thus been identified as the functional link between mTOR signalling and ribosome biogenesis [6].

It has been estimated that the human genome contains only 19,000–21,000 protein-coding genes but more than 70,000 proteins are synthesized [7]. This is explained by the alternative processing of pre-messenger RNA to create multiple mature mRNA transcripts; thus a single gene has the potential to encode many protein isoforms. This mechanism increases proteome complexity [8]. However, despite genes encoding multiple mRNA transcripts, often only one protein is dominantly expressed [9] indicating a process of selection or ‘censoring’ of mRNA that are successfully processed and released into the cytoplasm [10].

**Figure 1.**
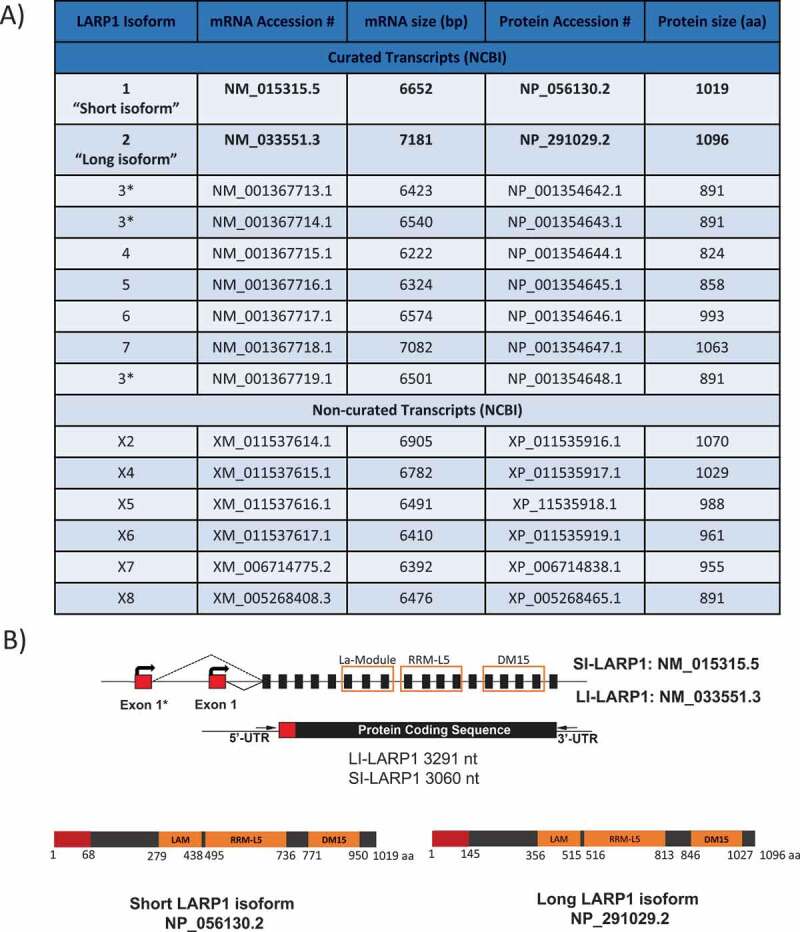
Isoforms of LARP1

The gene encoding human LARP1 is located at chromosome 5 (5q33.2) with at least nine known curated putative mRNA isoforms (https://www.ncbi.nlm.nih.gov/gene). Those isoforms are predicted to encode proteins ranging in size from 824 to 1096 amino acids (Fig. 1A). The ‘canonical’ LARP1 is listed on the The National Center for Biotechnology Information (NCBI) website as transcript variant 1 (NM_015315.5) encoding a protein of 1019 amino acids (NP_056130.2) known here as the ‘short isoform of LARP1’ (SI-LARP1). This is often used as the definitive LARP1 for studies of its function [6,11,12]. A second transcript variant 2 (NM_033551.3) is cited by UniProtKB as being the canonical form of LARP1 [1,2,13] and corresponds to a 1096 amino acid protein (NP_291029.2) referred to here as the ‘long isoform of LARP1’ (LI-LARP1). Each of these two LARP1 isoforms is transcribed from different promoter sites and has a unique transcription start site (TSS) resulting in a different exon 1 sequence whilst the remaining exons (exons 2–19) are identical. The resulting SI-LARP1 protein differs from LI-LARP1 by lacking the first 77 amino acids and with different amino acids M_1_ to N_67_.

A) Table of all reported LARP1 isoforms on NCBI (stand 04.11.2019). B) Representative illustration of the exon organization of ‘short’ and ”long” LARP1 isoforms and respective translated proteins. Both isoforms differ in nucleotide/amino acid sequence and length of exon 1 (red). Black squares represent exons. Line represents untranslated regions (UTR). La-module (LAM), RNA recognition motif L5 (RRM-L5) and LARP1 specific DM15 domain are given in orange. C) *In silico* analysis of expression levels of short and long LARP1 isoforms indicates a prevalent expression of the long isoform in primary cells, tissue and cancer cell lines. Data extracted from ENCODE database and visualized using UCSC Genome browser [23,44]. Also see Supporting Data 1 for a full list of analyzed data

Western blotting using antibodies raised to various regions of LARP1 generates a duplex band of approximately 150/130kDa, which has been interpreted as representing both LARP1 isoforms [12]. To explore this, we systematically conducted a series of *in silico*, transcriptomic, biochemical and cellular assays to elucidate the true expression profile of LARP1 in cancer and normal cells.

## Results

### Isoform landscape of LARP1 in cancer cells

**Figure 2.**
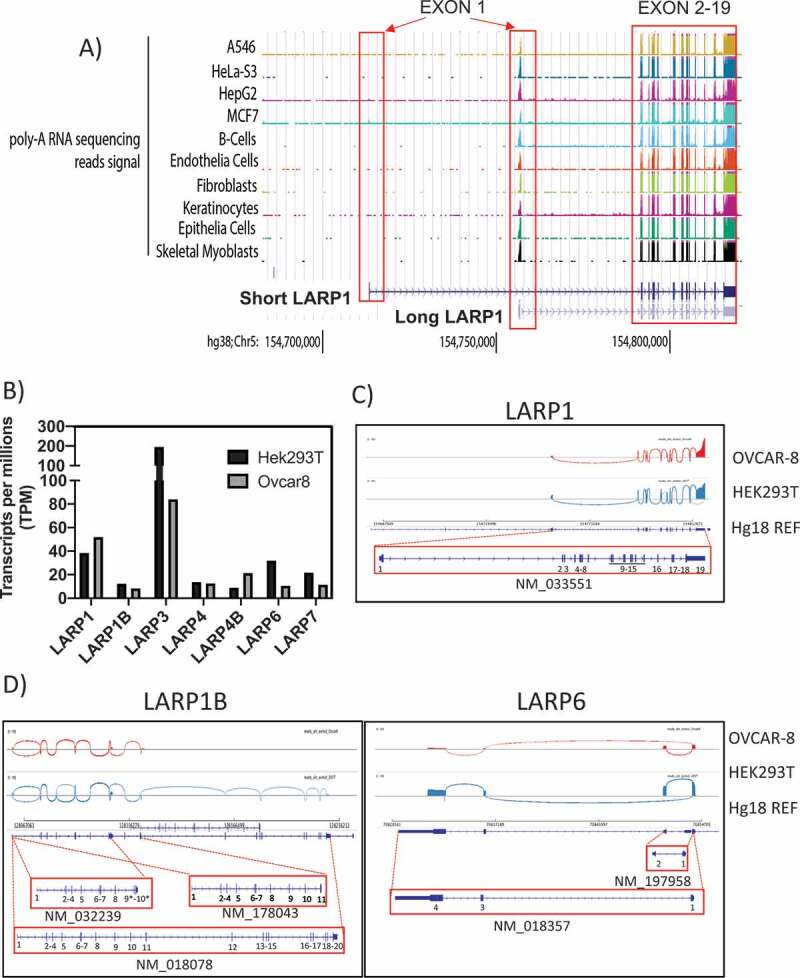
Transcriptomic profile of LARP1 isoform expression

Nine different validated isoforms are listed for *LARP1* in the NCBI gene database (Fig. 1A). Among those, the ‘canonical’ *LARP1* is listed as transcript variant 1 (NM_015315.5) encoding a protein of 1019 amino acids (NP_056130.2) known here as the ‘SI-LARP1’ (Fig. 1B). To investigate the expression profile of LARP1 in cells we used publicly available RNA sequencing datasets (Fig. 2A). We extracted 371 submitted biosamples containing poly-(A) RNA sequencing data sets from 198 tissues, 128 cell lines and 54 primary cells and mapped their reads to the chromosome region hg38:Chr5 154 700 000 to 154 850 000 containing all predicted LARP1 isoforms [14]. First, we focused on sequencing reads mapping to the first exon, which is distinct for both LARP1 isoforms. We found that the available sequencing data exclusively mapped to exon 1 of the long LARP1 isoform, with no reads mapping to exon 1 of SI-LARP1. Exons 2–19, which are shared between the two isoforms, were fully covered by the available sequencing data.

A) In silico analysis of expression levels of short and long LARP1 isoforms indicates a prevalent expression of the long isoform in primary cells, tissue and cancer cell lines. Data extracted from ENCODE database and visualized using UCSC Genome browser. Also, see Supporting Data 1 for a full list of analyzed data. B) Quantification of transcripts after direct RNA sequencing of OVCAR-8 and HEK-293 T cells by Nanopore Sequencing. C) Splice site selection of LARP1, D) LARP1B and LARP6 in OVCAR-8 and HEK-293 T cells. Sashimi plots are shown for most frequent splice site selections. A detection cut off of minimum of 2 exon-exon junction coverage was applied. The most frequently detected transcript variants are shown below the plot including the NCBI accession number. Also see Supporting Data 2.

To gain further insights into the expression of LARP1 isoforms we conducted Oxford Nanopore sequencing technology (ONT) in HEK-293 T and OVCAR-8 cells, immortalized human embryonic kidney and ovarian cancer cell lines, respectively (Fig. 2 B-D). This direct RNA sequencing technique allows the detection of long read transcripts isoforms and allows the quantification of transcript abundance without PCR bias [15]. ONT reads were mapped to human genome (Hg38) and aligned reads were visualized using the integrated genome viewer (IGV). In HEK-293 T cells, we detected a total of 2,038,842 transcripts while only 1,834,870 transcripts were detected in samples from OVCAR-8 cells. First, we quantitated all transcripts mapping to the seven LARP family members and normalized the reads to the total amount of detected long read transcripts (transcripts per million TPM) (Fig. 2B). We observed that, of the LARP family members, LARP3 has the highest number of transcripts at 194 TPM. In contrast, the other LARPs showed values well below 50 TPM with the expression of LARP1B and LARP4B being lowest. Interestingly, LARP1 shows a higher expression level in cancerous OVCAR-8 (52TPM) cells than in HEK-293 T cells (38TPM) in agreement with previous studies [4]. To analyze the expression of LARP1 isoforms in these cells, we visualized the splice junctions using Sashima plots (Fig. 2C). Using this direct sequencing approach, SI-LARP1 was undetectable in the two tested cells lines. Although expression of the LARPs was low compared to other genes e.g. GAPDH (up to 6396 TPM in OVCAR-8 cells)(Supporting Data 2), we clearly could identify LI-LARP1 as the predominantly expressed LARP1 isoform. Interestingly, we also observed an accumulation of 5ʹ truncated LARP1 transcripts. Of note co-expression of alternative isoforms was detected for LARP1B (NM-018078 NM_178043 NM-032239) and LARP6 (NM_197958, NM_018357) (Fig. 2D). For the other LARPs, we were able to confirm that the major transcript detected by ONT sequencing corresponded with the canonical isoform (Supporting Data 2).

**Figure 3.**
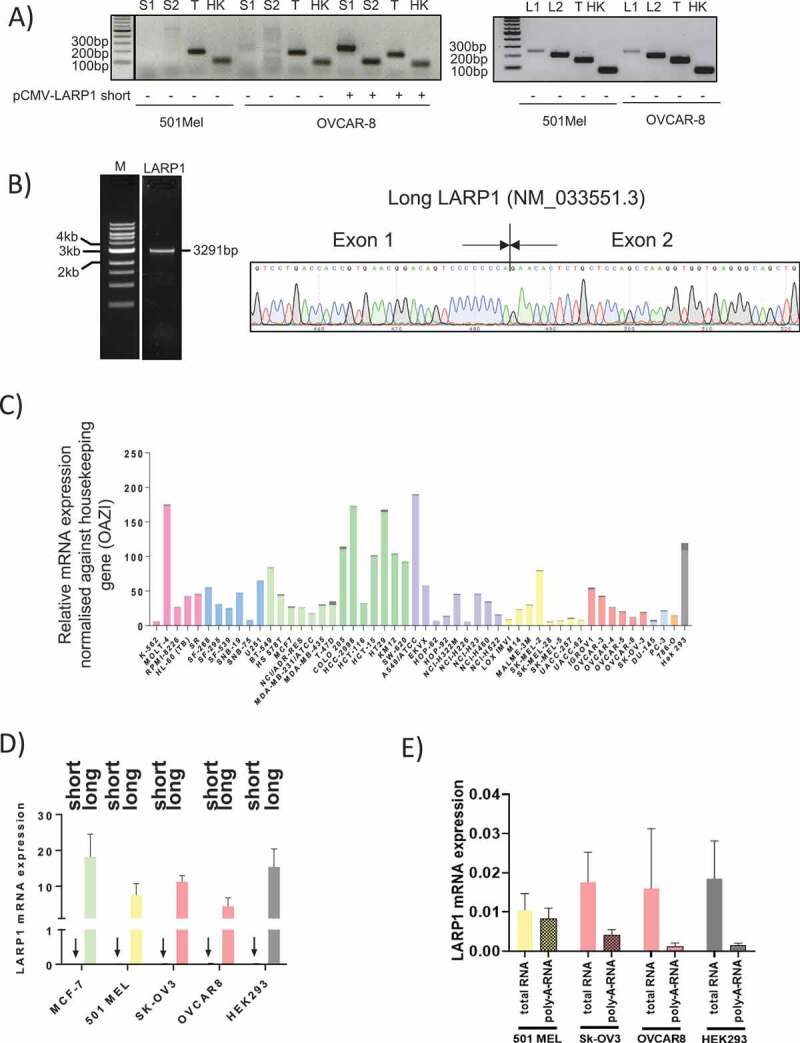
Analyzes of the mRNA expression of the short and long isoform in human cells

To validate our ONT sequencing findings that LI-LARP1 is the dominant isoform, we designed two pairs of specific primers to detect either the short or long LARP1 isoforms or both forms (referred to here as ‘total LARP1’) by PCR (Fig. 3). Forward primers corresponding to exon 1 of each LARP1 isoform and reverse primers to the exon junctions of downstream exons were designed. Functionality of these primers was confirmed by successful amplification following expression of recombinant SI-LARP1 or LI-LARP1. Primers for detecting total LARP1 were designed to anneal in exon 7, common to all predicted isoforms. We detected the expression of LI-LARP1 in 501-MEL and OVCAR-8 cells but we could not detect a PCR amplification product for SI-LARP1 (Fig. 3A). All PCR products were sequenced to validate the desired amplicon (data not shown). Next, we designed primers specific to the open reading frame of the long LARP1 isoform and amplified it by PCR from a cDNA library. Sequencing of the PCR product revealed the full predicted nucleotide sequence for LI-LARP1 including its specific exon 1 (Fig. 3B). Of note, all attempts to amplify the coding sequence of the SI-LARP1 isoform failed, indicating that this isoform did not exist in our cDNA library.

A) PCR amplification of ”short” and ‘long’ LARP1 isoforms using specific primers hybridizing to exon 1 of the respective isoform. S1/S2 and L1/L2 stand for primer pairs detecting SI- or LI-LARP1, respectively. T stands for total RNA detected using a primer pair annealing to constitutively expressed Exon 7. HK stands for housekeeping gene OAZ1. B) PCR product containing the complete open reading frame of long LARP1 was generated using gene-specific primers annealing to 5ʹ- and 3ʹ- end of the open reading frame. Amplification products were purified and sequenced. Junction of ‘long’ LARP1 specific exon 1 and common exon 2 is given in chromatograms. C) Systematic analysis of mRNA expression levels of short and long LARP1 isoforms in NCI-60 cancer panel using quantitative PCR. Specific primers for short and long LARP1 were used. Results of one representative primer pair for each isoform are shown. Dark and light-coloured bars represent proportion of SI- or LI-LARP1 expression, respectively. D) Expression of LI-LARP1 and absence of SI-LARP1 mRNA was validated in SK-OV-3, OVCAR-8, MCF-7, and 501-MEL cells using quantitative PCR. Resulting data were normalized against OAZ1. (E) Quantitative PCR analysis of expression of short LARP1 using random or oligo-(dT) primers for reverse transcription suggest that mRNA is missing poly-(A) tail. All qPCR data were normalized against OAZ1 and relative expression values were calculated using 2-Δct method.

### Long LARP1 isoform is dominantly expressed in NCI-60 cancer panel

To determine whether LI-LARP1 is expressed in a wider panel of cancer cell lines, we investigated the expression levels of LI-LARP1 and SI-LARP1 across the NCI-60 cancer cell panel [16] containing a selection of melanoma, renal, ovarian, brain, leukaemia, breast, non-small cell lung and prostate cancer cell lines (Fig. 3C). Although there was an up to 37-fold variation in expression of LI-LARP1 between the samples, it was detectable in all cell lines. However, mRNA expression of SI-LARP1 was below the threshold of detection in all lines (cT values <35). We validated these findings by measuring SI and LI-LARP1 in cell lines from the NCI panel (MCF-7, OVCAR-8, SK-OV-3, 501-MEL and HEK-293 T)(Fig. 3D). In all five cell lines, levels of LI-LARP1 were at least 1000 fold higher than of the SI-LARP1. Since expression levels of SI-LARP1 were close or below the detection limits, we explored whether the remaining detected transcripts were artefacts. A hallmark of mature mRNA is the existence of a 3ʹ poly-(A) tail, which is required for its nuclear export [17]. Polyadenylated total mRNAs, extracted from OVCAR-8, SK-OV-3, 501-MEL and HEK-293 T, were reverse transcribed using either random or oligo-(dT) primers (Fig. 3E). As random primers anneal to any type of transcript within the RNA pool, PCR amplification will detect also non-processed mRNA species. In contrast, oligo-(dT) primers lead to a specific reverse transcription of polyadenylated mRNAs. Levels of SI- and LI-LARP1 isoform in both cDNA libraries were measured using gene-specific primers. Expression levels were normalized against OAZ1 and relative mRNA expression was calculated. We found that levels of fully processed and polyadenylated SI-LARP1 mRNA were below detection limits suggesting the SI-LARP1 mRNA is expressed but not fully processed to mature, polyadenylated mRNA.

Altogether, our data indicate that the mRNA for the long LARP1 isoform is the dominantly expressed form in wide range of cancer and normal cells and that the SI-LARP1 mRNA, if expressed, is present at low levels and in an immature form.

### Identification of peptides specific for long isoform in four different human cell lines

**Figure 4.**
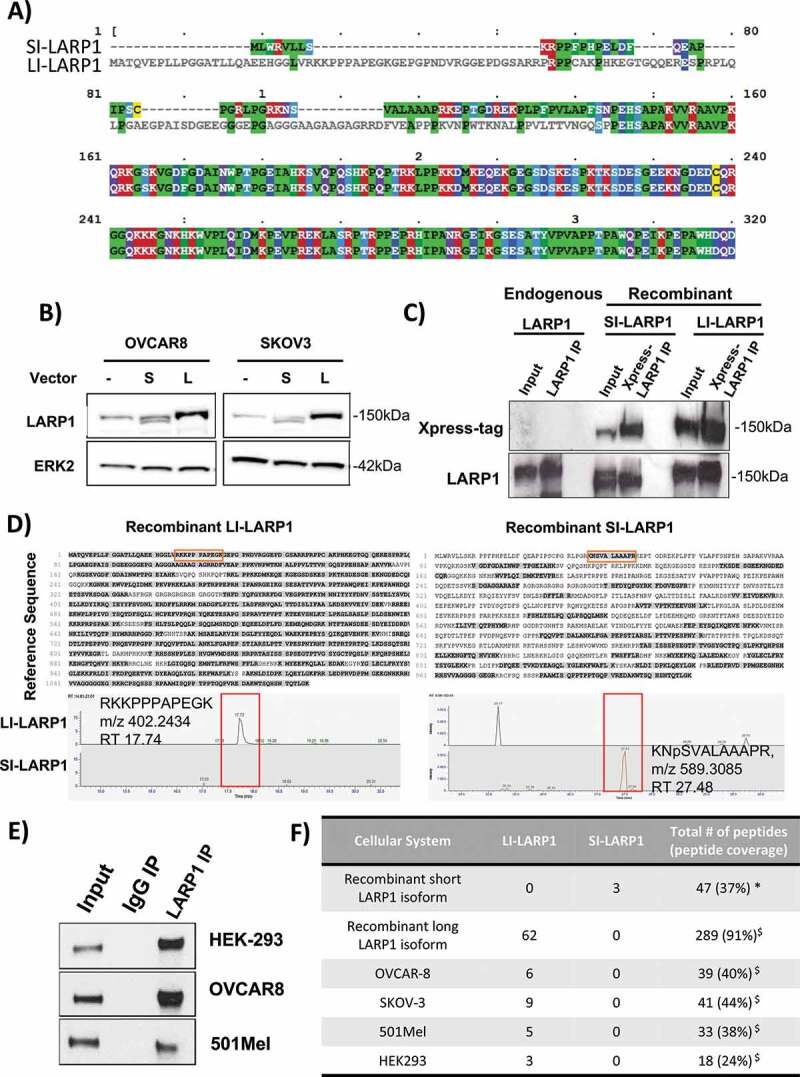
Identification of peptides specific for long isoform in four different human cell lines

As described previously, SI- and LI-LARP1 have distinct N-termini encoded by different exon 1 sequences (Fig. 4A). Both exons end in-frame, thus the exon selection does not affect the downstream reading frame and amino acid composition of LARP1. The long isoform constitutes a 77 amino acid longer polypeptide chain and leads to protein predicted to be 8.4 kDa larger. When both forms are exogenously expressed only a small difference in the migration pattern on an SDS-Gel was observed (Fig. 4B). Comparing the migration patterns of exogenously expressed SI- and LI-LARP1 with endogenous LARP1 showed the latter co-migrates with LI-LARP1 indicating that the protein product of LI-LARP1 is dominantly expressed. To validate this, we followed a two-step strategy; first we ectopically expressed both LARP1 isoforms in OVCAR-8 cells and enriched the LARP1 protein by immune-precipitation from crude protein lysate using antibodies against the co-expressed Xpress tag (Fig. 4C and Supporting Data 3). Subsequently, samples were subjected to *in solution-digestion* using Trypsin or Chymotrypsin. Shotgun LC-MS/MS analyses was performed to identify isoform-specific peptides mapping to the N-terminus of either the SI-or LI-LARP1 isoform (Supporting Data 4). We identified three peptides generated from the sequence position at 45 and 46 (with masses of N_46_SVALAAAPR: 485.2773 Th (m/z); K_45_NpSVALAAAPR: 549.3257 Th (m/z); K_45_NSAVALAAAPR: 589,3085 Th (m/z)) as unique to the SI-LARP1 (Fig. 4D). In contrast, for the LI-LARP1 we identified 152 peptides generated from the protein sequence corresponding to exon 1 (Supporting Data 4). In a second step, endogenous LARP1 from OVCAR-8, 501-MEL, SK-OV-3 and HEK-293 T cells was immuno-precipitated and subjected to mass spectrometry to detect pre-specified isoform-specific peptides using a supervised and automated algorithm (Fig. 4 E-F). The peptide coverage for LARP1 ranged from 24-44% for the endogenous immunoprecipitated protein. Although unique peptides corresponding to LI-LARP1 N-terminus were identified in all four cell lines, we could not identify any peptides specific to SI-LARP1 isoform in any of the samples. These results are consistent with the observed absence of mRNA corresponding to the SI-LARP1.

A) A sequence alignment of N-terminal protein sequence of SI-and LI-LARP1 protein. B) Detection of endogenous and recombinantly expressed SI- and LI-LARP1 protein using antibody raised against peptide common to both isoforms. For expression constructs see Supporting Data 3. C) To identify the peptides corresponding to LARP1, we immunoprecipitated endogenous and overexpressed SI- and LI-LARP1 isoform using either LARP1 or Xpress tag specific antibodies. Detection of total ERK2 served as loading control. D) Immunoprecipitated samples were subjected to *in solution* tryptic or chemotryptic digestion and mass spectrometric analysis were performed. Resulting peptides were mapped to either long or short LARP1 protein sequence (grey shadow). Mass spectrometry identified peptides mapping specifically to short or long LARP1 isoform, when ectopically expressed (red square). MS/MS Spectra, m/z and retention time (RT) for peptides specific to SI- or LI-LARP1 are given. E) Control western blots of LARP1 pull downs using specific antibodies against LARP1. IgG control is shown. F) Summary table from peptide identification specific to LARP1 by mass spectrometry from recombinant and endogenous LARP1. Number of identified peptides specific for SI-or LI-LARP1 are given. Total number of peptides and peptide coverage for SI-(*) and LI-LARP1 ($) is given.

### Promoter of short isoform is hypermethylated which suppresses its promoter activity

**Figure 5.**
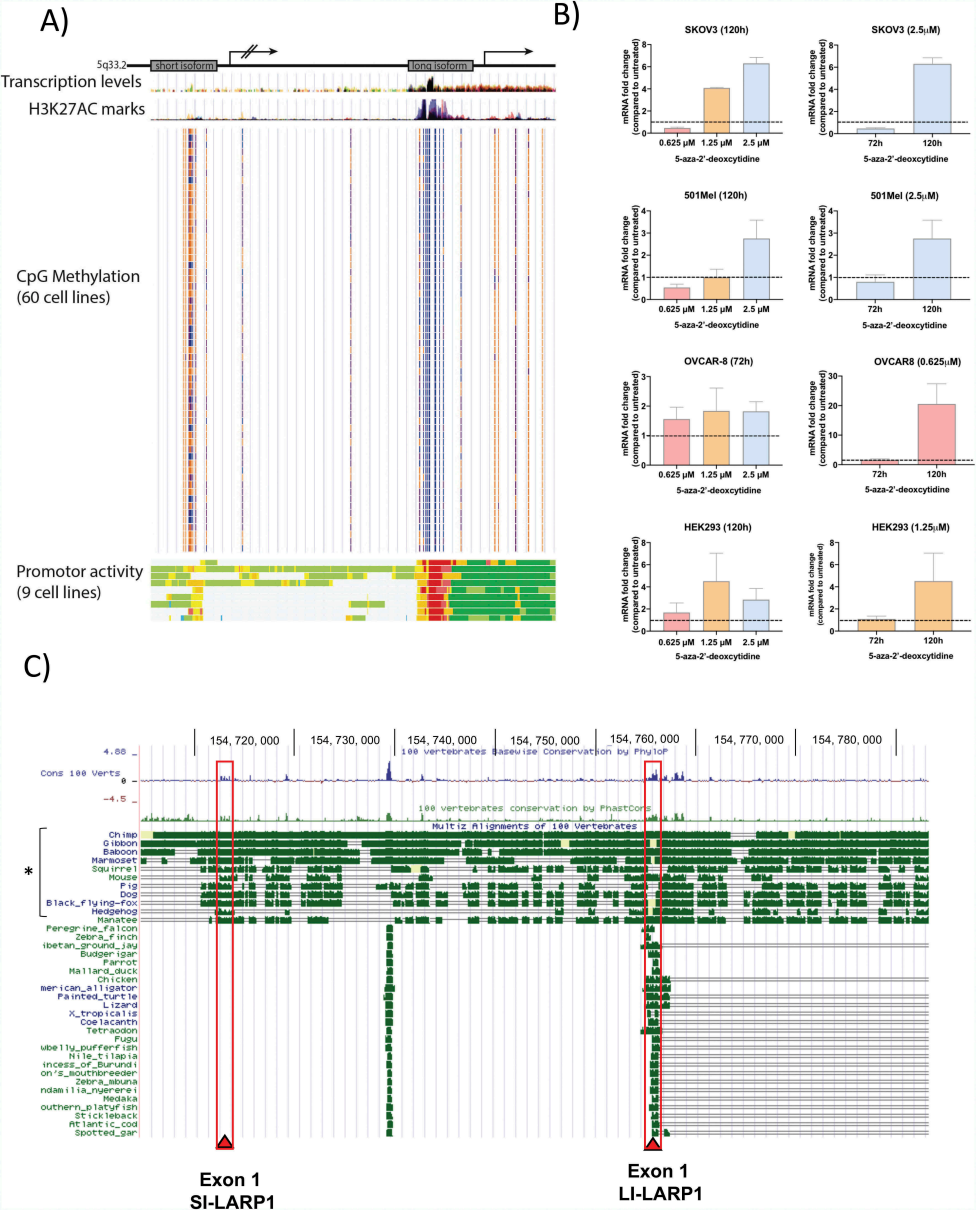
Promoter of long isoform is demethylated and activated while promoter of short isoform is methylated and inactive

Next, we investigated the mechanism by which expression of the SI-LARP1 is regulated. Transcriptional activity correlates with chromatin state, which is characterized by histone modifications, open chromatin and specific transcription factor binding [18]. Therefore, we used three publicly available datasets to predict the promoter activity of the short and long LARP1 isoforms (Fig. 5A). The predicted TSS of the SI-LARP1 is located at GRCh38.p13:154,712,843 while the TSS of the LI-LARP1 is within the first intron of the SI-LARP1 at position GRCh38.p13:154,755,377. We judged the transcriptional activity based on three parameters. First, we used ChIP-seq data to identify H3K27ac marks, which represents acetylation of lysine 27 of H3 histone protein leading to transcription enhancement [19]. Second, we used data from human methylation arrays to identify CpG methylation, which correlates with silencing of genes and, conversely, their demethylation stimulates gene expression [20,21]. Third, we predicted the chromatin state segmentation for nine human cell lines by computationally integrating ChIP-seq data using a multivariate Hidden Markov Model (HMM) [22]. All data were aligned to GRCh37/hg19 and analyzed using the UCSC genome browser (Fig. 5A) [23]. We found high levels of CpG methylation but low H3K27AC marks within the SI-LARP1 TSS suggesting diminished promoter activity. In contrast, the LI-LARP1 TSS had low levels of CpG methylation and high H3K27AC marks suggesting an active promoter site. Of note for SI-LARP1, the only significant increase in promoter activity was observed in human embryonic stem cells. Altogether our data indicate that the promoter region of LI-LARP1 is in an open chromatin state, suggesting a high promoter activity for this region while the promoter of the SI-LARP is epigenetically repressed.

A) *In silico* analysis of H3K27AC marks, CpG methylation and promoter activity of SI- and LI-LARP1. CpG Methylation is indicated by a heatmap. Red represents the highest and blue for the lowest methylation. Short isoform promoter activity is predicted to be a weak enhancer (yellow) while the region around the long isoform promoter comprises an active promotor (red) with strong enhancer elements and transcriptional transition and elongation sites. B) Quantitative PCR analysis of SI-LARP1 of 5ʹ Azacytidine treated cells at different concentration over 3 days or fixed concentration at different timepoints, using random primers for reverse transcription were performed. C) *In silico* analysis of the conservation of exon 1 of the short and long LARP1 within vertebrates. Conservation is calculated by Phylop or Phastcons and represented as blue or green peaks, respectively. Multiple sequence alignment is shown from representative organisms of the vertebrate subphylum (mammals, amphibians, reptile, fish and bird). The mammalian subdivision placentalia is highlighted with a bracket (*). Location of exon 1 of short and long isoform is indicated by red frame. Pairwise alignments of each species to the human genome are displayed and nucleotide conservation is indicated by green bars. Data have been extracted from UCSC genome browser [23].

To confirm the repression of the SI-LARP1 promoter by CpG methylation, we sought to re-activate its expression by demethylation (Fig. 5B) using 5ʹ Azacitidine (5-Aza), a chemical analogue of cytidine and an inhibitor of DNA methyltransferase [24]. To do so, SK-OV-3, OVCAR-8, 501-MEL and HEK-293 T cells were treated with an increasing concentration of 5-Aza for 3 or 5 days. To confirm the re-activation of expression of SI-LARP1, we measured the levels of SI-LARP1 mRNA in treated and control cells by quantitative PCR (Fig. 5B) and observed up to fivefold concentration-dependent increase in SI-LARP1 after 3 days. When we used an optimized concentration and increased the incubation time up to 5 days, we found a 20-fold increase of SI-LARP1 mRNA in OVCAR-8. However, we did not detect any protein corresponding to the SI-LARP1 by western blotting (Supporting Data 5B). Of note, we did not observe a significant increase in LI-LARP1 mRNA nor protein expression (Supporting Data 5A-B). These data indicate that expression of SI-LARP1 is to some extent epigenetically regulated. However, even when epigenetically de-repressed, SI-LARP1 is not synthesized into protein by the cytoplasmic translation machinery.

Next, we explored whether the expression pattern of LI-LARP1 is similar to that of both SI-LARP1 and endogenous LARP1. Previous work by Hopkins et al. showed that transient knowdown of LARP1 using siRNA targeting both SI-LARP1 and LI-LARP1 in OVCAR-8 and SK-OV-3 cells was associated with a decrease in cell viability and increase apoptosis[4]. To validate that this observation is attributable to LI-LARP1, we targeted mRNA of LI-LARP1 with specific siRNAs to unique exon 1 or the ubiquitous exon 13 (Supporting Data 5 C). Both siRNAs caused a reduction in cell viability when compared to OVCAR-8 cells treated with scramble siRNA (Supporting Data 5 C). Interestingly, the lower protein band (that measures approximately 130 kDa) in the LARP1 western blot duplex is successfully depleted using siRNA against exons 13, but not using siRNA against its N-terminal exons (Supporting Data 5C). This indicates the band corresponds to an unknown N-terminally truncated variant of LARP1.

Furthermore, it is well established that LARP1 interacts with PABP to bring the 5ʹ and 3ʹ UTR of the target mRNA in close vicinity [25,26]. To prove this was true for LI-LARP1, we pulled down endogenous and ectopically expressed LARP1 and found that both SI-LARP1 and LI-LARP1 were able to bind PABP (Supporting Data 5D).

Next, we were interested whether SI-LARP1 could have a distinct functional role. To investigate this, we analyzed the level of conservation of TSS of both LARP1 isoforms (Fig. 5C). We hypothesized that evolutionary conservation would hint towards a selective pressure and consequently a different role for each isoform. To decipher the evolutionary conservation of the LARP1 isoforms we generated a multiple sequence alignment of the 100 available LARP1 sequences in subphylum of vertebrates (Birds, Fish, Amphibia, Reptiles, Mammals) using UCSC genome browser [23]. Surprisingly, we found a strong conservation of exon 1 of LI-LARP1 through the whole subphylum but conservation of SI-LARP1 exon 1 was only present in the Placentalia subdivision, which includes humans and other primates. This indicates it may have acquired a functional role late in evolution, specific to placental mammals. By contrast, the conservation of LI-LARP1 indicates a far more ancient role across multiple clades.

## Discussion

Although LARP1 is a known post-transcriptional regulator, its dominant isoform has been disputed. We show here that mRNA and protein corresponding to the long isoform (or LI-LARP1) is ubiquitously expressed in cancer and non-cancer cells. In contrast, transcripts encoding SI-LARP1 could be only detected when the promoter was demethylated suggesting it is, to some extent, epigenetically regulated. However, we were unable to detect a protein product following epigenetic derepression, indicating a more sophisticated post-transcriptional regulation [27]. Importantly, we show that the lower band of the 150/130 kDa duplex commonly observed in western blots of endogenous LARP1 (and attributed to its long and short isoforms) [4,12] infact does not represent a protein product of SI-LARP1. However, the 130 kDa band appears to be an N-terminally truncated variant of LARP1 rather than a product of degradation. The function of this as yet unknown LARP1 variant has yet to be determined.

Our findings support a recently published study predicting over 10 potential alternative promoters for LARP1 [28] and are consistent with evidence that most coding genes have multiple isoforms but only one expressed protein [9]. Whilst the evolutionary conservation of an expressed isoform indicates it has a conserved biological function [29], the role of non-expressed isoforms remains controversial [9,30]. While some hypothesize they are transcriptional noise [31], others propose they are required to regulate protein function, interactions or localization[32] or to generate an evolutionary testing ground allowing functions and domains to evolve [33,34]. Indeed, we show that exon 1 of SI-LARP1 is highly conserved within the mammalian Placentalia subclade suggesting it provides a functional and thus evolutionary advantage distinct from LI-LARP1. Furthermore, detection of SI-LARP1 mRNA only in human embryonic stem cells, may indicate a role in embryonic development. The exact role of SI-LARP1 and subcellular localization needs to be addressed in future research.

The first exons from SI- and LI-LARP1 map to a highly unstructured region upstream of the La-Module that does not contain distinct protein domains or known substrate binding regions [13]. This is consistent with the observation that alternative exons locate to sequences within intrinsically disordered protein regions rather than functional domains [35,36]. As these regions are sites of post-translational modification and involved in protein–protein interactions [37–39], it has been speculated that remodelling of intrinsically disordered domains expands the cell-type or condition-specific gene repertoire [39].

We show that variation in isoform expression is observed in other LARP family members. Using long read RNA sequencing, we identified several alternatively expressed isoforms of LARP1B and LARP6. Both express a full length and a significantly shorter isoform. Interestingly, their expression also seems to differ between cancer and non-cancer cell lines. Indeed, it has been previously shown that cancer associated splicing changes can drive tumour progression [39]. It would be interesting to investigate whether those isoforms encode differentially expressed proteins that fulfil distinct molecular functions.

In general, RBPs like the LARPs are commonly recognized as ‘cytoplasmic transcription factors’, that bind and regulate the stability, half-life and subcellular localization of multiple RNAs and are mobilized in response to cellular stress [40]. Using currently available sequencing tools, we show that there is significant censoring of LARP1 expression in favour of a single isoform that is predominantly expressed. This provides an intriguing insight into the evolution and functional relevance of LARP1 and the wider LARP family of proteins.

## Materials and Methods

### Cell culture and transfections

SK-OV-3, 501-MEL and OVCAR-8 cells were cultured in RPMI medium with 10% (v/v) *Foetal Bovine Serum* (FBS) and 1% (v/v) Pen Strep. HEK-293 T and MCF-7 cells were cultured in DMEM with high Glucose, GlutaMAX™, 10% (v/v) FBS and 1% (v/v) Pen Strep. For primer validation, OVCAR-8 cells were transfected with 1 μg of pCMV6 plasmid containing the coding sequence for the short or long isoform (Supporting Data 3) using FuGene6 transfection reagent, following manufacturers instructions. After 24 h incubation, the cells were washed in PBS, harvested and RNA extracted. For LARP1 knockdown, OVCAR-8 cells were transfected with Lipofectamine RNAiMax (ThermoFisher) and siRNAs targeting LARP1, either ‘Exon 13’ (AGACUCAAGCCAGACAUCA) or ‘LI-LARP1 Exon 1’ (GUGAACCCGUGGACUAAGAAC). Cells were trypsinized 24 h after transfection and reseeded for proliferation assays. Cyquant NF Cell Proliferation assay (ThermoFisher) was used to monitor the quantity of viable cells at 24 h intervals, with values normalized against initial readings taken after cells had adhered. Concurrently, cell lysates were harvested 48 h after siRNA transfection for Western blotting.

### Nanopore sequencing and analysis

OVCAR-8 and HEK-293 T cells were seeded in 10 cm dish and grown overnight to 70% confluency. Cells were washed with PBS and RNA was extracted using Trizol [41]. Poly-(A)RNA was captured using Oligotex mRNA mini kit (Qiagen) according to manufactures instructions. RNA was directly sequenced using Direct RNA sequencing kit SQK-RNA002 (Oxford Nanopore) according to manufactures instructions. Libraries were sequenced on R9.4.1 flow cells which were run for 8 h using live basecalling, files were outputted in Fast5 and Fastq format. Fastq files were concatenated and processed through the Oxford Nanopore Pinfish RNA analysis pipeline (https://github.com/nanoporetech/pipeline-pinfish-analysis). In brief, this pipeline aligns reads to the GRCh38 reference genome and then identifies, polishes and collapses transcripts into a consensus GFF file.

### In silico analysis

RNA expression levels of LARP1 were analyzed using next generation sequencing data extracted from ENCODE project (Extraction date 27.09.2018). We downloaded 371 call sets from the ENCODE portal [40] containing Poly-(A) RNA sequencing data from 198 tissues, 128 cell lines and 54 primary cells. Unique positive strand reads were mapped to either long (XM_005268404.3) or short (NM_015315.4) isoform of LARP1 using UCSC Genome browser on Genome Reference Consortium Human Build 38 patch release 12 (GRCh38.p12) human GRCh38/hg38 assembly (Dec 2013) [23].

CpG methylation as determined by Illumina Infinium Human Methylation 450 Bead Array (ENCODE project) aligned to GRCh37/hg19 was selected to be displayed in dense mode within the UCSC genome browser. The dataset comprised over 63 different cell lines.

Due to absence of annotation of the long isoform of LARP1, the genomic location was extracted from GRCH38/hg38 XM_005268404.3 at chr5:154755575–154817605 and NM_015315.4 at chr5:154712902–154817607 for the short and long isoform, respectively. Genome coordinates were converted into GRch37/hg19 genome annotation using the LiftOver tool at UCSC browser.

Third, we predicted the chromatin state segmentation for nine human cell lines by computationally integrating ChIP-seq for nine-factors plus input using a multivariate Hidden Markov Model (HMM) [22].

### RNA extraction, cDNA synthesis and quantitative PCR

RNA extraction was performed according to manufacturer's protocol of the GeneElute^TM^ Mammalian Total RNA Miniprep Kit (Sigma-Aldrich, Cat. No. RTN70-1KT) and a DNase digestion was performed to reduce genomic contamination using RNase-Free DNase Set from QIAGEN.

One μg RNA was reverse transcribed using High-Capacity cDNA Reverse Transcription Kit (Applied Biosystem). If not otherwise stated, random primers were used. Twenty μl of cDNA reaction was diluted with 130 μL RNase-free water and stored at −20°C until further use.

The quantitative polymerase chain reaction (qPCR) was carried out to analyze the cDNA samples in a MicroAmp^Ⓡ^ Fast 96 well plate reaction plate (Applied Biosystems) using 2x Fast SYBR Green Master Mix (Thermo Fisher Scientific). Quantitative PCR was performed in three technical replicates. For the run, the StepOnePlus Real-Time PCR System (Applied Biosystems) was used with the fast run mode (40 min) alongside melting curve analyzis. Full length LI-LARP1 cDNA was generated using Maxima Reverse Transcriptase (Thermo Scientific) and oligo-(dT) primers. cDNA was amplified by PCR using Qiagen LongRange Polymerase with primers amplifying the full length coding sequence (CDS). PCR product was purified and cloned into a pCR4-TOPO vector following the instructions of the TA-Cloning^TM^ kit (Invitrogen). Insert of purified plasmid DNA was sequenced (Eurofins).

### Analysis of NCI-60 cDNA library

The cDNA of the NCI-60 human cancer cell panel [16] was kindly provided by Gareth Bond (Ludwig Institute for Cancer Research, Oxford). A 1:20 dilution of the cDNA was performed by mixing 5 μL of the cDNA sample and 95 μL nuclease free water. The screen was performed by qPCR analysis using the following seven primer pairs: OAZ1, GAPDH, LARP1 long 1, LARP1 long 2, LARP1 short 1, LARP1 short 2. Primers were selected for amplification efficiency of 100% (± 10%) and specificities were validated using melting curve analyses. Quantitative PCR analysis was performed using Fast SYBR Green Master Mix (Thermo Fisher Scientific) using the fast amplification protocol according to manufacturer's instructions. The average of three replicates was normalized against either OAZ1 or GAPDH housekeeping genes and relative mRNA abundance was calculated using the 2 – ^Δct^ method [42].

### Primers

Following primer pairs were used for the analysis: LARP1 short 1 CTTTGGAGGGTGCTTTTGTC, AGTGTTCAGGGTTGCTGAAG; LARP1 short 2 CCAGAGCTGGATTTCCAAGA, CTAAACAGCGCAAAGGCAG; LARP1 long 1 GTGAACCCGTGGACTAGAAC, ACTGTGGCTGAACACTCTTG; LARP1 long 2 CCCAGGAACACTCTGCTC, ACTGTGGCTGAACACTCTTG; total LARP1 GAACCCATTTTGACTACCAG, TTGATGTAGTCTTTGAGGCAG; OAZ1 ATAGTCAGAGGGATCACAATC, CGTTAGTTCCTCTGTTACATTC; GAPDH GTCTCCTCTGACTTCAACAGCG, ACCACCCTGTTGCTGTAGCCAA; CDS ATGGCCACTCAGGTGGAGC, TCACTTTCCCAAAGTCTGTGTGTTC

### Overexpression and Pull-down of short and long isoforms of LARP1

OVCAR-8 cells were cultured in 10 cm dish and transfected with 2 µg of pcDNA 4 constructs expressing Hismax/Xpress-tagged codon-optimized coding sequences of short (NM_015315.4.1) and long (XM_005268404.3.1) isoforms of LARP1 using Fugene 6 transfection reagent (Promega) according to the manufacturer’s instructions. Forty-eight hr following transfection, cells were lysed using ice-cold immunoprecipitation (IP) lysis buffer (50 mM Tris HCl, pH 7.6, 150 mM NaCl, 20 mM MgCl_2_ and 0.5% NP-40) supplemented with EDTA-free protease and phosphatase inhibitors cocktail (Roche). Cell lysates were incubated for 30 min with agitation at 4°C, followed by centrifugation at 13,000 rpm for 15 min. Resulting supernatants were then collected, and protein concentration was determined using Pierce Coomassie Plus (Bradford) Assay Reagent (Life Technologies). For each sample, an aliquot of cell lysate (10%) was saved as input protein and the rest was incubated with 20 µg of anti-LARP1antibody (ab86359, Abcam) or Xpress Antibody (R910-25, Thermo Fisher Scientific) at 4°C overnight with agitation. 70 µl of Dynabeads Protein G (Life Technologies) was added to each IP sample and incubated at 4°C for 2 h with agitation. Dynabeads-Ab-Ag complexes were then washed 3 times by gentle pipetting using 100 µl of washing buffer (PBS pH 7.4, 0.02% Tween 20). LARP1 immunoprecipitates were eluted from beads by heating samples in 1% sodium dodecyl sulphate (SDS)/β-mercaptoethanol at 95°C for 5 min, then loaded onto NuPAGE 3-8% Tris-acetate gels (EA0378BOX, Life Technologies) for immunoblotting.

### Mass spectrometry

*In-solution* digest, SDS containing samples were precipitated with Chloroform/Methanol twice [43] after dilution up to a volume of 175μl and reduction/alkylation with DTT and Iodoacetamide. Protein pellets were re-suspended in 100μl 6 M Urea, diluted with 500μl water before digest with 0.6μg Trypsin or Elastase over night. Samples were acidified and desalted using SOLA cartridges (Thermo Pierce).

Peptides were injected into a nano LC-MS/MS platform consisting of a Dionex Ultimate 3000 UPLC and an Orbitrap Fusion Lumos mass spectrometer. Peptides were separated on a 50 cm nEASY Spray column (ES803A) with a gradient from 5-35% Acetonitrile in 5%DMSO/0.1% formic acid over 60 min. MS- spectra were acquired at a mass resolution of 120000 and an AGC target of 400000. Precursors were isolated with a mass window of 1.6Th and fragmented by HCD. Peptide fragments were analysed in the Orbitrap at 30000 resolution and an AGC target of 50000 for up to 54 ms. Collision energy was set to 30%. Precursors were acquired with the top speed methods and a 3 s cycle time and then excluded from repeated selection for 7 s.

MS/MS data were analysed with PEAKS 8 (Bioinformatics Solutions). Mass tolerances were set to 5ppm (precursor) and 0.03 Da (fragment). Carbamidomethyl (Cys) was set as fixed modification and Oxidation (M), Deamidation (Asn, Gln) and Phosphorylation (Ser, Thr, Tyr) were set as variable modifications. The used database (Uniprot reviewed, Homo sapiens, retrieved 26/06/2018) was supplemented with known LARP1 isoforms (see Fig. 1). Peptide FDR was adjusted to 1%. Extracted ion chromatograms of isoform-specific LARP1 peptides were generated with Freestyle (Thermo).

### Genome-wide demethylation

The demethylation of the genome was carried out by a 5-Azacytidine treatment of the following four cell lines: SK-OV-3, 501-MEL, OVCAR-8 and HEK-293 T cells. Briefly, cells have been cultivated in a 10 cm cell culture dishes to 70% confluency. Indicated concentration of 5-Azacytidine (ab142744, Abcam) was added to the cells and medium was replaced daily. Control cells were treated with a respective volume of DMSO. Cells were harvested after 3 and 5 days, washed with PBS and total RNA was extracted. Messenger RNA expression was analyzed by qPCR.

## Supplementary Material

Supplemental Material
