# Supplementary material for: LARP1 isoform expression in human cancer cell lines
Source: RNA Biol. 2020 Apr 14;18(2):237–47. doi: 10.1080/15476286.2020.1744320 (PMC7928056; doi:10.1080/15476286.2020.1744320)
Supplement: Supplemental Material [file KRNB_A_1744320_SM8888.zip › Supplementary information/Supporting Data.docx]

**Supporting Data:**

**Supporting Data 1: Transcriptomic profile of LARP1 isoform expression in primary cells, tissue and cancer cells**

In Silico Analysis of expression levels of short and long LARP1 isoforms indicates a prevalent expression of the long isoform in cancer cell lines. Extended data related to Figure 2A. Total and Poly-A RNA sequencing data from different primary cells (A), tissue (B) and cancer cells (C). Each track represent the plus strand signal of unique reads for the genomic location of LARP1 gene. LARP1 gene transcript organisation is given at the bottom. Tracks are sorted and colored by biosamples. All call sets were downloaded from ENCODE portal (<https://www.encodeproject.org>), visualized using UCSC Genome browser and identifiers datasets are given.

**Supporting Data 2**: **Direct RNA sequencing of OVCAR-8 and HEK-293T cells by Nanopore Sequencing**.

Read alignment of Oxford Nanopore Sequencing to the Human Genome (hg18). Read coverage, splice junction and reference sequence are given for all seven LARP family members. In addition, the most frequently detected transcript variants are shown and NCBI transcript accession numbers are given.

**Supporting Data 3: Vector constructs used as positive control for expression of SI- and LI-LARP1.** CMV- cytomegalovirus promoter, 6x-His - hexa histidine-tag, Xpress - DLYDDDDK Xpress tag peptide sequence. General transfection and pulldown flow chart is given.

**Supporting Data 4:** **Identification of peptides specific for long isoform in four different human cell line.**

Mass spectrometry of recombinant expressed A) LI-LARP1 and B) SI-LARP1 protein. Mapping of identified peptides (blue bars) to SI- and LI-LARP1 reference protein sequence. Identified peptides used to distinguished SI- or LI-LARP1 are highlighted by red square. C) MS/MS spectra and D) annotation table for isoform specific peptide is given.

**Supporting Data 5**: **Functional characterisation of LI-LARP1**

A) Quantitative PCR analysis of LI-LARP1 in 5’ Azacytidine treated cells at different concentration over 3 days or 5 days at indicated concentration, using random primers for reverse transcription were performed. B) Western Blot analysis of 5’Azacitidine treated cells using a antibody specific to LARP6 and loading control GAPDH. Time points and concentrations are given. C) Knockdown of endogenous LARP1 using siRNA targeting ubiquitinous exon 13 (total LARP1) or LI-LARP1 specific exon 1. Asterix indicates the additional uncharacterised 130 kDa LARP1 specific band. Cytotoxity assay indicates percentage of viable cells after knockdown. D) To identify proteins interacting with LARP1, endogenous and overexpressed SI- and LI-LARP1 isoform were immune precipitated using either LARP1 or Xpress-Tag specific antibodies. PABP1 specific antibody was used. # Same experiment as in Figure 4 C.
