# Supplementary figures and images for: LARP1 isoform expression in human cancer cell lines
Source: RNA Biol. 2020 Apr 14;18(2):237–47. doi: 10.1080/15476286.2020.1744320 (PMC7928056; doi:10.1080/15476286.2020.1744320)

## LARP1

293T

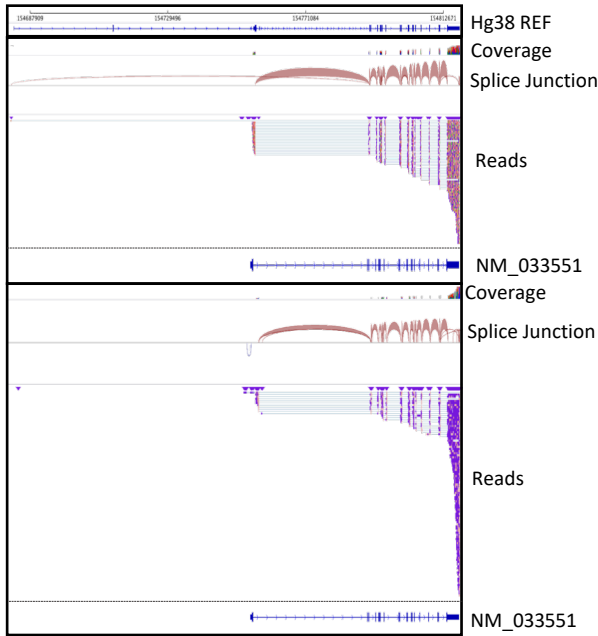

Ovcar 8

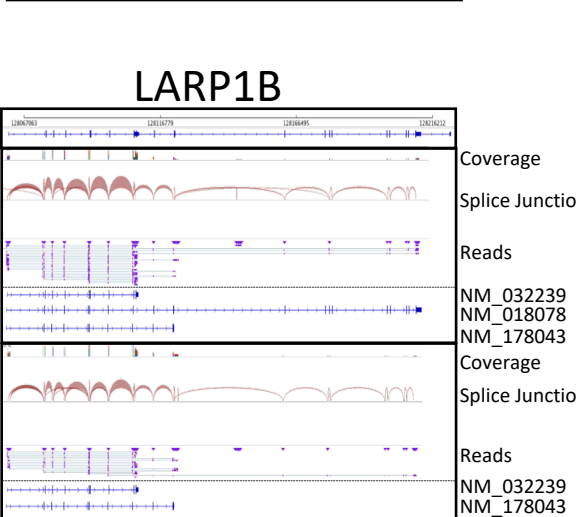

## LARP1B

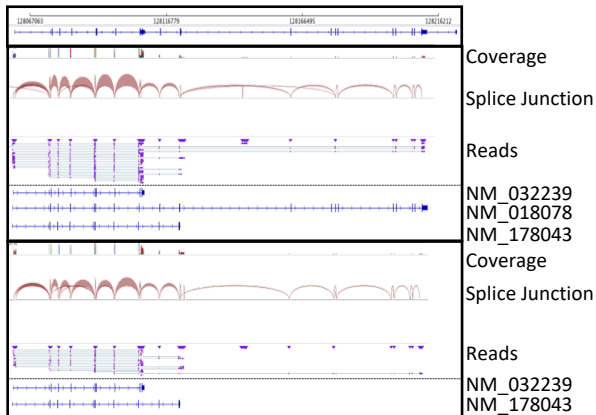

## SS-B/LARP3

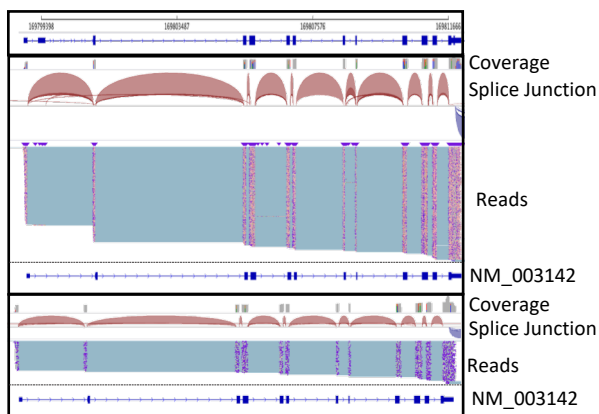

## LARP4

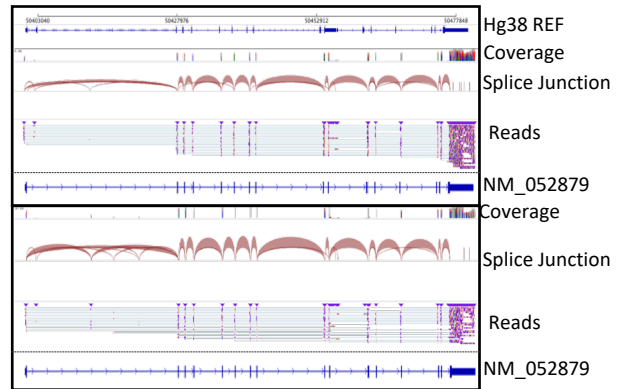

## LARP4B

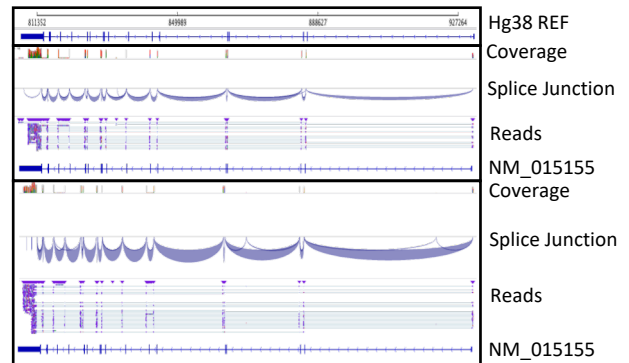

## LARP6

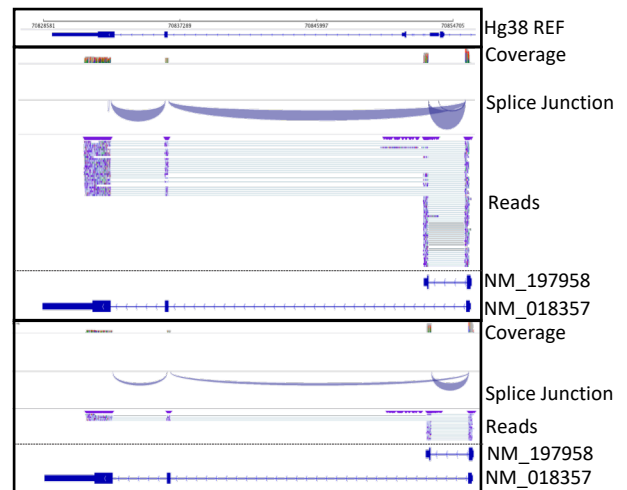

## LARP7

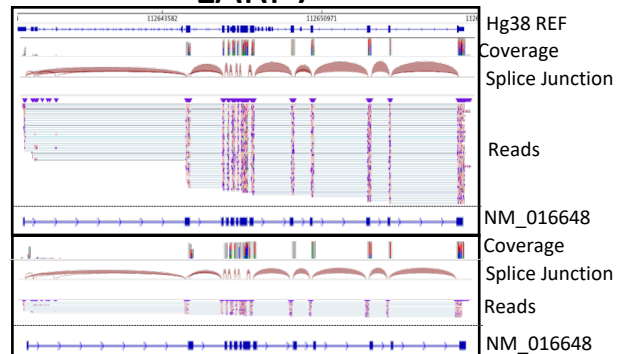

Supplement: Supplemental Material [file KRNB_A_1744320_SM8888.zip › Supplementary information/Supporting Data 2.pdf]

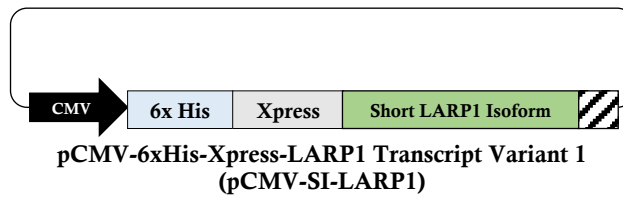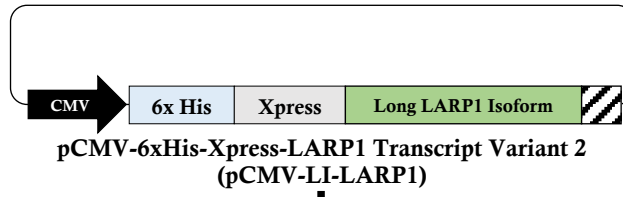

Individually transfected into OVCAR8  
(48 hr)

Pull-down of Xpress-tagged LARP1

Supplement: Supplemental Material [file KRNB_A_1744320_SM8888.zip › Supplementary information/Supporting Data 3.pdf]

## SI-LARP Reference Sequence

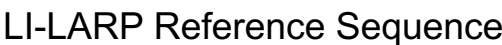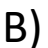

## SI-LARP Reference Sequence

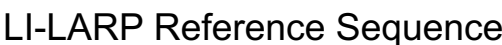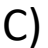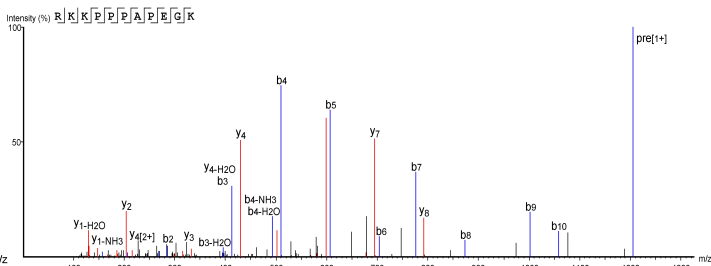

D)

| #  | b       | b-H2O   | b-NH3   | b (2+) | Seq | y       | y-H2O   | y-NH3   | y (2+) | #  |
|----|---------|---------|---------|--------|-----|---------|---------|---------|--------|----|
| 1  | 157.11  | 139.10  | 140.08  | 79.05  | R   |         |         |         |        | 11 |
| 2  | 285.20  | 267.19  | 268.18  | 143.10 | K   | 1048.61 | 1030.60 | 1031.59 | 524.81 | 10 |
| 3  | 413.30  | 395.29  | 396.27  | 207.15 | K   | 920.52  | 902.51  | 903.49  | 460.76 | 9  |
| 4  | 510.35  | 492.34  | 493.32  | 255.68 | P   | 792.42  | 774.41  | 775.40  | 396.71 | 8  |
| 5  | 607.40  | 589.39  | 590.38  | 304.20 | P   | 695.37  | 677.36  | 678.35  | 348.19 | 7  |
| 6  | 704.45  | 686.45  | 687.43  | 352.73 | P   | 598.32  | 580.31  | 581.29  | 299.66 | 6  |
| 7  | 775.49  | 757.48  | 758.47  | 388.25 | A   | 501.26  | 483.26  | 484.24  | 251.13 | 5  |
| 8  | 872.54  | 854.54  | 855.52  | 436.77 | P   | 430.23  | 412.22  | 413.20  | 215.62 | 4  |
| 9  | 1001.59 | 983.58  | 984.56  | 501.29 | E   | 333.18  | 315.16  | 316.15  | 167.12 | 3  |
| 10 | 1058.61 | 1040.60 | 1041.58 | 529.81 | G   | 204.13  | 186.12  | 187.11  | 102.57 | 2  |
| 11 |         |         |         |        | K   | 147.11  | 129.10  | 130.09  | 74.06  | 1  |

Supplement: Supplemental Material [file KRNB_A_1744320_SM8888.zip › Supplementary information/Supporting Data 4.pdf]

A)

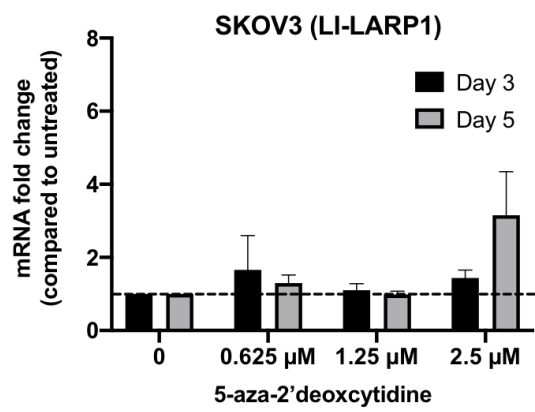

B)

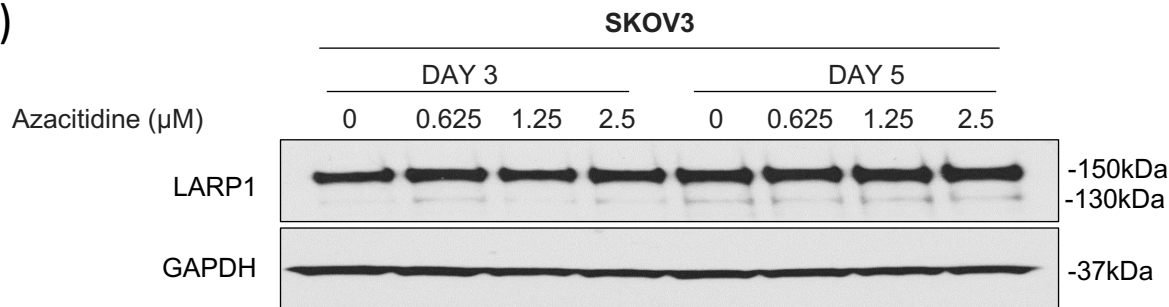

C)

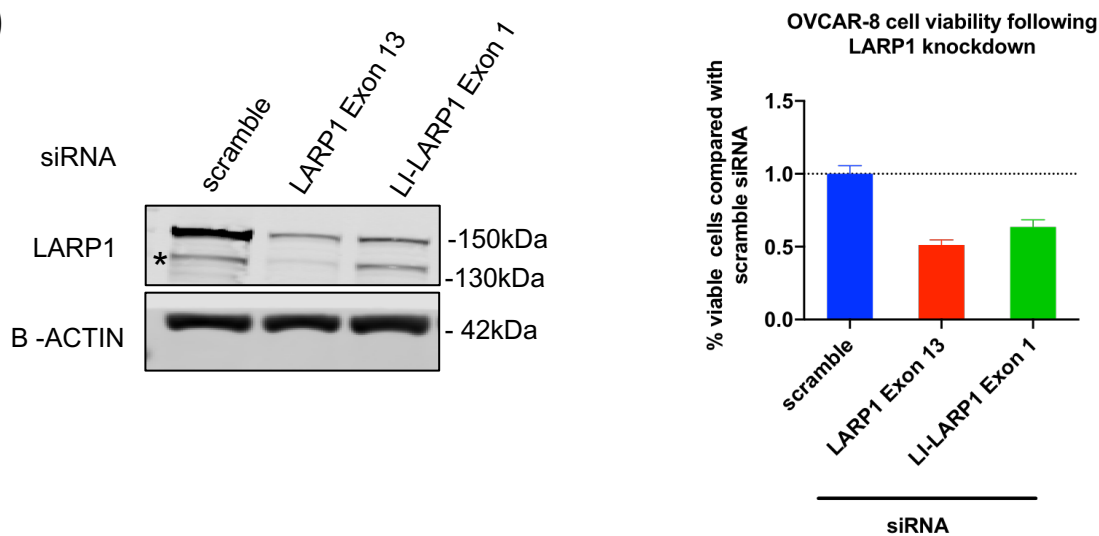

D)

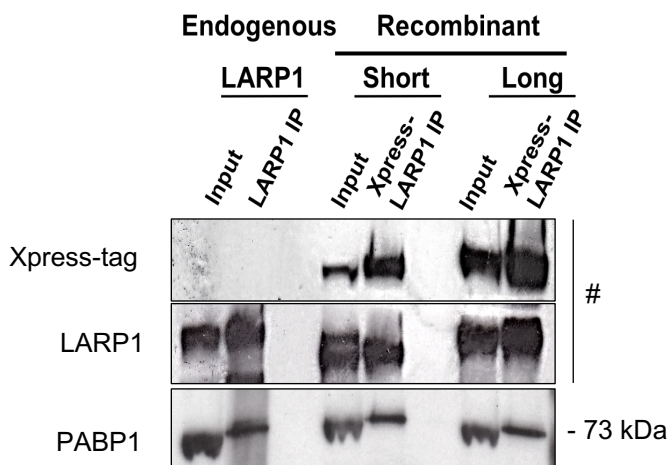

Supplement: Supplemental Material [file KRNB_A_1744320_SM8888.zip › Supplementary information/Supporting Data 5.pdf]
